# Pediatric Neuroimaging of Multiple Sclerosis and Neuroinflammatory Diseases

**DOI:** 10.3390/tomography10120149

**Published:** 2024-12-20

**Authors:** Chloe Dunseath, Emma J. Bova, Elizabeth Wilson, Marguerite Care, Kim M. Cecil

**Affiliations:** 1 Medical School, University of Cincinnati College of Medicine, University of Cincinnati, Cincinnati, OH 45267, USA; 2 Department of Radiology, Cincinnati Children’s Hospital Medical Center, Cincinnati, OH 45229, USA; 3 Department of Pediatrics, Cincinnati Children’s Hospital Medical Center, University of Cincinnati College of Medicine, University of Cincinnati, Cincinnati, OH 45229, USA; 4 Department of Radiology, University of Cincinnati College of Medicine, University of Cincinnati, Cincinnati, OH 45219, USA

**Keywords:** demyelination, neuroinflammatory, magnetic resonance imaging, magnetic resonance spectroscopy, multiple sclerosis, acute demyelinating encephalomyelitis, myelin oligodendrocyte glycoprotein antibody disease

## Abstract

Using a pediatric-focused lens, this review article briefly summarizes the presentation of several demyelinating and neuroinflammatory diseases using conventional magnetic resonance imaging (MRI) sequences, such as T1-weighted with and without an exogenous gadolinium-based contrast agent, T2-weighted, and fluid-attenuated inversion recovery (FLAIR). These conventional sequences exploit the intrinsic properties of tissue to provide a distinct signal contrast that is useful for evaluating disease features and monitoring treatment responses in patients by characterizing lesion involvement in the central nervous system and tracking temporal features with blood–brain barrier disruption. Illustrative examples are presented for pediatric-onset multiple sclerosis and neuroinflammatory diseases. This work also highlights findings from advanced MRI techniques, often infrequently employed due to the challenges involved in acquisition, post-processing, and interpretation, and identifies the need for future studies to extract the unique information, such as alterations in neurochemistry, disruptions of structural organization, or atypical functional connectivity, that may be relevant for the diagnosis and management of disease.

## 1. Introduction

Magnetic resonance imaging (MRI) plays an integral role in the identification and management of medical conditions. Brain and spine MRIs are essential for characterizing suspected demyelinating and neuroinflammatory diseases and deriving a diagnosis, but each may also provide information for predicting prognoses and evaluating responses to therapy. A wide array of clinical and imaging literature describes patient findings throughout the evolution of signal abnormalities associated with these pathologies over the course of the disease. However, many existing reviews are limited to case presentations with neuroimaging of adult patients.

Pediatric presentations of these diseases can be non-specific and follow a course that differs from adults, especially with respect to MRI features. We sought to conduct an overview style of review to conceptually summarize the literature without a formal quality assessment, given the relatively small amount of literature in this area. We compiled a list of seven pediatric diseases encountered at our institution over the past 5 years, where neuroimaging was illustrative. We searched PubMed (pubmed.ncbi.nlm.nih.gov) for each disease with the term “magnetic resonance imaging”. We performed subsequent analyses with the disease and the specific advanced magnetic resonance imaging modalities. We extracted articles with all or a majority of pediatric populations. In a few instances with advanced resonance imaging in rare conditions, we highlighted general disease features independent of the population age. Upon reviewing these articles, citations of conceptual interest from the articles were also incorporated into the bibliography. We also searched PubMed for each disease with the terms “incidence” and “prevalence”. We also included published literature review articles to help us condense the salient findings and provide a resource for readers. This work highlights key aspects from the literature on the incidence, clinical presentation, and conventional MRI findings for multiple sclerosis (MS), acute demyelinating encephalomyelitis (ADEM), optic neuritis (ON), neuromyelitis optica spectrum disorders (NMOSD), myelin oligodendrocyte glycoprotein antibody disease (MOGAD), autoimmune encephalitis, and febrile-infection-related epilepsy syndrome (FIRES). Also, insights are shared from the relatively limited usage of advanced MRI techniques, such as proton magnetic resonance spectroscopy (MRS), magnetization transfer imaging (MTI), diffusion-weighted imaging (DWI) including diffusion tensor imaging (DTI), and functional MRI (fMRI), in pediatric populations. These techniques can be added to a conventional MRI examination to provide distinct yet complementary information to clarify the disease features.

## 2. Multiple Sclerosis

### 2.1. Overview

Pediatric-onset multiple sclerosis (POMS) involves an immune-mediated attack on the central nervous system (CNS), resulting in inflammation, demyelination, and axonal damage [1,2]. There are a variety of typical neurological symptoms in children, such as vision changes accompanied by ON, sensory symptoms, gait impairment, and cognitive deficits [1,3,4,5]. The diagnosis of POMS follows the revised McDonald 2017 adult criteria for MS, which requires two distinct clinical events without encephalopathy lasting at least 24 h and occurring more than 30 days apart that feature lesions in an MRI disseminated in space and time [6,7,8]. Among all demyelinating and neuroinflammatory diseases, MS is one of the most common [9]. However, POMS is relatively rare [10,11], constituting 3–5% of all patients with MS [3,5,12,13,14]. A meta-analysis published in 2020 reported the pooled global incidence and prevalence estimates for POMS as 0.87 per 100,000 individuals annually and 8.11 per 100,000 individuals, respectively [15]. POMS has the highest incidence rates in children between the ages of 13 and 16 and occurs more often in females post-puberty [5]. Patients with POMS almost exclusively follow a relapsing–remitting course and tend to have a higher relapse frequency, a higher lesion burden, especially with contrast-enhancing lesions, and an earlier age at disability milestones compared to adult-onset MS [3,5,6,11,16,17,18].

Upon presentation, it can be difficult to distinguish the first demyelinating episode of POMS from other demyelinating diseases or disease processes (vasculitis, neoplasm, infection, infarction, etc.). An MRI performed in the clinical setting is sensitive to disease pathology, which will narrow the diagnostic differential. Interpretation of MRI relies on identifying characteristics of pathologies via changes in the signal contrast. A conventional MRI incorporates and exploits the intrinsic properties of water (and other tissues such as fat) to achieve a signal contrast with different sequences. The sequence nomenclature (T1-weighted, T2-weighted, etc.) reflects the nature of the property exploited.

### 2.2. Conventional Neuroimaging

**Figure 1:**
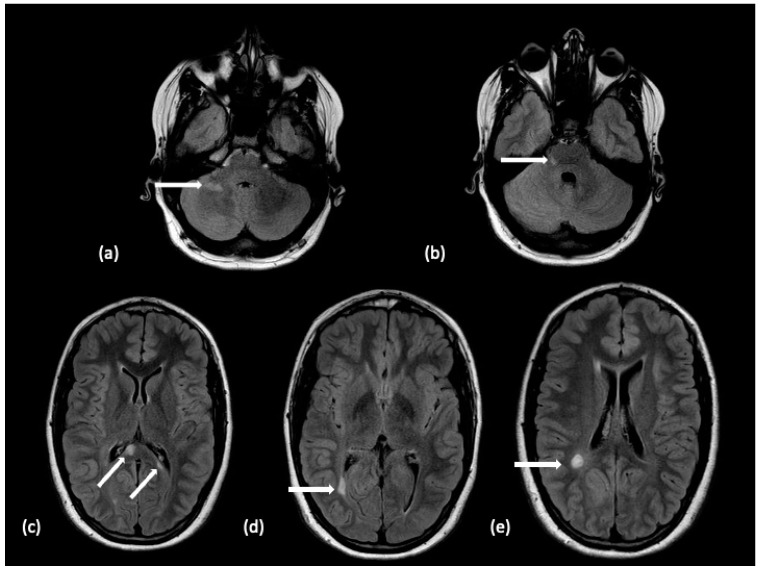
A 14-year-old male with a 3-year history of multiple demyelinating, non-enhancing brain lesions. A follow-up MRI demonstrated an increase in both size and number of lesions with a distribution within the brain characteristic of pediatric-onset multiple sclerosis. Non-enhancing lesions on an axial FLAIR sequence without diffusion restriction are noted with white arrows within the (**a**) right hemisphere cerebellar white matter, (**b**) dorsal right pons, (**c**) splenium of the corpus callosum, (**d**) bordering the right occipital horn, and (**e**) right hemispheric parietal white matter. The features supported the diagnosis of pediatric-onset multiple sclerosis.

In POMS, conventional MRIs, including T1-weighted, T2-weighted, and fluid-attenuated inversion recovery (FLAIR) sequences, reveal periventricular lesions in the deep white matter, juxtacortical lesions, infratentorial lesions, and spinal cord lesions spanning less than three vertebral segments [19,20,21]. Abnormalities on T2-weighted and FLAIR sequences in POMS characteristically appear as focal ovoid-shaped hyperintensities [22]. One characteristic imaging finding of MS is Dawson’s Fingers, described as well-defined ovoid lesions aligned perpendicular to the long axis of the corpus callosum on sagittal MRI, whose presence increases the rate of a second attack [4,20,23]. White matter lesions with hypointense signals relative to normal-appearing white matter (NAWM) on T1-weighted sequences are referred to as “black holes” [24]. Exogenous contrast agents with chelated gadolinium are intravenously injected to reveal any blood–brain barrier disruption in MRI examinations. Gadolinium contrast agent signal enhancement, which may be present for approximately 3 weeks in MS lesions, assists in differentiating between active/recent lesions from inactive/older lesions [22,25]. Examples of typical brain lesions found in POMS are illustrated in Figure 1. Critical diagnostic markers indicative of an initial demyelinating event progressing to MS include the presence of Dawson’s fingers, T1 hypointense lesions (black holes), contrast-enhancing lesions, or periventricular lesions on the MRI [23,26].

**Figure 2:**
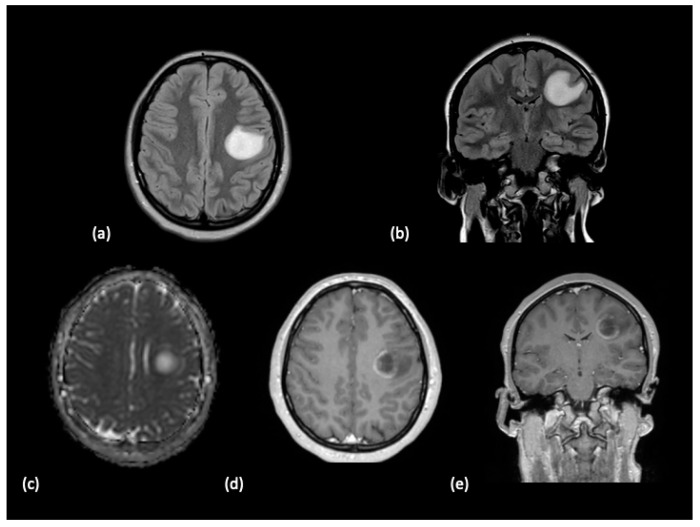
A 15-year-old female with a history of bilateral optic neuritis presented with stroke-like symptoms including facial droop and slurred speech. (**a**) Axial and (**b**) coronal FLAIR sequences demonstrated a large hyperintense lesion located in the left middle frontal lobes, precentral gyrus and the frontal operculum. (**c**) The lesion exhibits mild diffusion restriction at the periphery. Partial peripheral enhancement along the medial margin of the lesion was noted on the (**d**) axial and (**e**) coronal post-contrast T1-weighted images. The patient history and imaging features over time supported a diagnosis of tumefactive multiple sclerosis.

**Figure 3:**
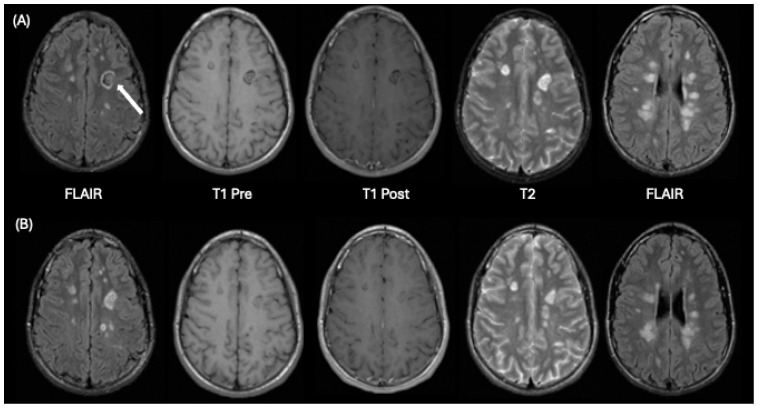
A 14-year-old male who presented with a history of headaches and vague numbness in his right upper limb. Row (**A**) demonstrates initial baseline imaging and Row (**B**) shows follow-up imaging 8 months later. From left to right in each row are FLAIR, T1-weighted pre-contrast, T1-weighted post-contrast, T2-weighted axial images at the level of a concentric ring lesion (white arrow) in the left frontal lobe and a FLAIR image at a superior level illustrating periventricular lesions. This appearance is consistent with Balo’s concentric sclerosis. Comparison of corresponding images in rows A and B, show many lesions were smaller at follow-up imaging than on the prior baseline study. None of the patient’s lesions significantly enhanced after contrast administration at either timepoint on T1-weighted imaging.

**Figure 4:**
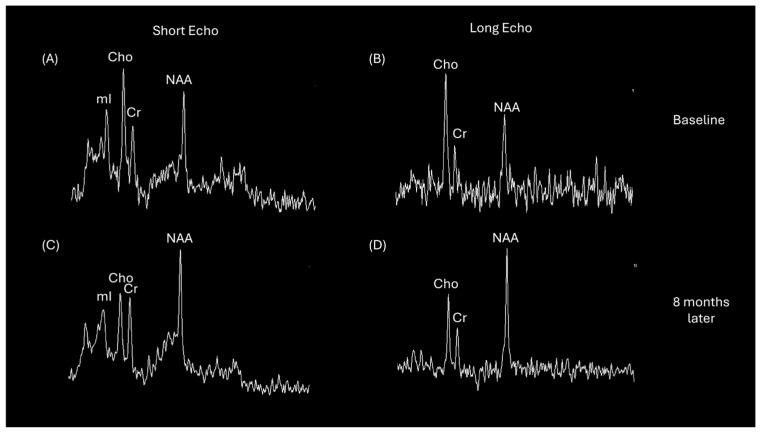
Short echo (35 milliseconds) and long echo (288 milliseconds) single voxel proton MRS acquired in the left frontal lobe Balo’s lesion of a 14-year-old male at initial baseline and follow-up examination 8 months later for the patient described in Figure 3. Short echo MRS in (**A**) demonstrates elevated choline-to-creatine and myo-inositol-to-creatine ratios compared with ratios observed in (**C**). Long echo MRS in (**B**) also reflects the choline elevation. The spectra normalize as shown in (**C**,**D**) with lower levels of choline and higher N-acetyl-aspartate.

One imaging variant of MS is tumefactive MS, with estimates that up to 40% of pediatric patients have tumefactive lesions [23,27,28]. These lesions are typically large and solitary, exhibiting a mass effect and ring enhancement, which can lead to misdiagnosis as a brain abscess or a neoplasm [20,27,29]. Figure 2 illustrates the initial presentation of a tumefactive lesion in a patient with a history of ON. Another imaging variant of MS is Balo’s concentric sclerosis, where concentric rings of demyelination alternate with areas of preserved myelination, which are often visible on T2-weighted and post-contrast T1-weighed images [30]. Gadolinium contrast agent enhancement may demonstrate a breakdown of the blood–brain barrier within the center of the lesion, contrasting typical MS with rim enhancement [31]. Baló lesions can present at onset as a solitary lesion or multiple lesions in white matter but are typically absent in cortical U-fibers [32]. While Balo’s concentric sclerosis is considered rare, there are several recent pediatric case presentations in the literature [30,31,32,33,34]. Figure 3 and Figure 4 illustrate imaging and proton MRS obtained in a patient with Balo’s lesions.

In addition to classic white matter lesions, patients with POMS also exhibit pertinent gray matter findings on MRI. A longitudinal study evaluating regional and whole brain volumes multiple times over a 2-year period revealed that patients with POMS did not achieve age-expected primary brain growth and were accompanied by a greater reduction in the age-expected thalamic volume that is also associated with the T2 lesion volume [35]. Compared to sex- and age-expected trajectories, patients with POMS exhibit a significant reduction in gray matter development in several cortical regions, the cerebellum, and subcortical regions, including the thalamus and caudate nucleus [36]. Gray matter atrophy in patients with POMS correlates with clinical disability severity [36], consistent with findings in adults with MS [37,38]. Such longitudinal cohort analyses reveal unique information missed with an individual review of patient imaging. Group analyses of quantitative metrics derived from advanced MRI techniques offer the opportunity for new mechanistic insights.

### 2.3. Advanced MRI Techniques

While T1-weighted, with and without a gadolinium contrast agent administered, T2-weighted, and FLAIR are conventional neuroimaging sequences utilized to distinguish POMS apart from other diseases, there are advanced MRI techniques available to provide unique information to improve the specificity of MRI. MRS, MTI, and DTI may afford additional insight toward characterizing disease pathology. MRS provides novel information regarding brain neurochemistry with tissue metabolite profiles. Although research using MRS in patients with POMS is limited, findings have shown similar patterns to those observed in adults. Lesions demonstrate decreased N-acetyl aspartate (NAA) and creatine (Cr) alongside increased choline (Cho), myoinositol (mI), and lipid concentrations [39]. NAA, a neurochemical present in neurons and axons, serves as a marker of integrity and functioning and declines in correspondence with functional impairments during active MS phases. Choline concentrations reflect the status of cellular membranes, with increases observed during active demyelination. Myo-inositol is highly concentrated in astrocytes and functions as an osmolyte [40,41]. Consistent with demyelination as well as gliosis, mI concentrations within lesions are elevated in patients with MS [42,43,44,45]. MRS can also facilitate the assessment of recovered myelination in MS lesions following treatment, as NAA levels in acute lesions have been found to partially recover in parallel with the improvement of the patient’s clinical status [46,47,48], as illustrated in Figure 4. A study of adult patients found that MRS can assist in discriminating tumefactive MS lesions from high-grade glioma based on a comparison of Cho/NAA levels [49].

MTI facilitates quantification of small-scale damage within lesions visible on T2, as well as in NAWM, and normal appearing gray matter (NAGM) by providing a unique image contrast based upon detecting signals from macromolecules derived from proteins, lipids, carbohydrates, and nucleic acids [50,51]. Demyelinating lesions in patients with POMS are often hypointense on magnetization transfer ratio (MTR) maps [52]. MTI can also be used to monitor remyelination in patients with POMS over the course of treatment, as demyelinated lesions have a lower MTR than remyelinated lesions [52,53]. Compared to adults with adult-onset MS, adults with POMS show a lower MTR value in T2 lesions with hyperintense signals, NAWM, and NAGM [54]. This difference may be explained by the longer duration of disease in adults with POMS compared to those with onset later in life as adults [52,54].

DTI provides a quantitative method for assessing cerebral white matter microstructure damage by detecting changes in water diffusion, which is quantified by decreased fractional anisotropy (FA), increased mean diffusivity (MD), and changes in the apparent diffusion coefficient (ADC) [55,56,57]. Patients with POMS have lower average FA values and a widespread increase in MD measurements in the normal-appearing brain compared to healthy controls [52,55,56,57,58]. Patients with POMS also show reduced FA in NAWM and the corpus callosum, indicating disruption of the myelin architecture, even outside of areas with clear demyelinating lesions [55,56,59,60,61]. ADC is elevated in pathological processes when neuronal tissue damage removes barriers to water diffusion throughout the CNS [62]. Vishwas et al. found elevated mean ADC values throughout the white matter, including the NAWM of patients with POMS compared to healthy controls, suggesting evidence of diffuse damage early in the disease course that may be independent of disease duration [55].

Diffusion tensor metrics may afford additional objective and quantitative measures to monitor the extent of white matter damage and the impact on neurological function in patients. DTI studies of patients with POMS have been combined with fMRI examinations to better characterize disruptions in the functional connectivity (FC) of brain networks, which have implications for cognitive impairment and disability [52,63,64]. fMRI studies are acquired in one of two approaches: (1) task-dependent fMRIs are performed in coordination with the patient performing an activity in response to stimuli, such as viewing a visual checkerboard, responding to a continuous performance task, or performing other paradigms; (2) a resting state fMRI is acquired while the patient is not performing a specific task but often stares at a fixed point on a screen without other stimuli. A study of patients with POMS combining DTI with a resting state fMRI reported lower FA throughout the cerebral white matter and a higher FC within the intrinsic default mode and frontoparietal networks upon comparison with healthy controls, suggesting compensatory activation early within the disease [63].

While there are minimal disruptions in motor FC networks in patients with POMS [65,66], Rocca et al. found a distributed pattern of FC abnormalities within large-scale neuronal networks upon conducting resting state fMRI [67]. They found reduced FC in posterior brain regions of several sensory- and cognitive-related networks with increased FC in anterior brain regions. In contrast, adults with MS have an unevenly distributed pattern of FC variations [67,68]. The discrepancies between adult and pediatric findings may be secondary to disease duration and activity or represent a maturation deficit that renders certain developing brain regions more vulnerable to disease processes [67]. Longitudinal studies with appropriate statistical power incorporating DTI and resting state fMRI across the lifespan for POMS patients are needed to better characterize these maturational changes and potentially identify features that would be amendable to therapies.

While it is beyond the scope of this review, there are also many studies evaluating the cognitive abilities of patients with POMS and their relationships with imaging outcomes from structural MRI, diffusion MRI, and fMRI evaluations [52,61,64,69,70,71].

### 2.4. Summary of Imaging in Pediatric Patients with POMS

Patients with POMS almost exclusively follow a relapsing–remitting course and tend to have a higher relapse frequency and a higher lesion burden of contrast-enhancing lesions on T1-weighted imaging. T2-weighted and FLAIR sequences are sensitive to detecting lesions in the brainstem, cerebellum, subcortical white matter at the cortex, and periventricular lesions in deep white matter, especially those termed Dawson’s fingers. The quantitative nature of advanced MRI techniques holds relevance for future therapeutic trials as patients with POMS demonstrate metrics that differ from healthy controls, though their current usage often explores mechanistic features of the demyelinating process and provides characterization of the patient with temporal features of lesions.

## 3. Acute Demyelinating Encephalomyelitis

### 3.1. Overview

ADEM is a typically monophasic, immune-mediated neuroinflammatory condition most commonly occurring in children between 3.6 and 8 years old following a prior infection or vaccination [72,73,74,75,76,77], with an incidence of 0.3 to 0.6 per 100,000 population per year [74,78]. ADEM can occur as a single incident or more rarely (<10%) as the initial demyelinating attack that precedes MS or another relapsing demyelinating condition [26,79]. Patients typically present 1–2 weeks post-infection or vaccination with an acute onset of encephalopathy, polyfocal neurologic deficits, and demyelinating CNS lesions on conventional MRI [80]. Clinical symptoms include encephalopathy, pyramidal long tract signs, hemiplegia, ON, cranial nerve palsies, hyperreflexia, ataxia, seizures, meningism, and spasticity, which were preceded by several days of fever, vomiting, headache, and nausea [19,73,74,75,76,77,80,81,82]. Despite the severe clinical vignette, most patients with ADEM experience a significant recovery within weeks, although residual cognitive impairments or learning disabilities can occur [83,84].

If there are no additional clinical symptoms or MRI lesions at follow-up 3 months after the initial onset, the diagnosis of ADEM is confirmed [80,85,86]. It is important, however, to assess for secondary etiologies and risk factors for relapse. For example, myelin oligodendrocyte glycoprotein (MOG) immunoglobulin-G antibodies (MOG-IgG) are detected in 50% of pediatric ADEM cases, and those seropositive experience more relapses than seronegative cases [87].

### 3.2. Conventional Neuroimaging

**Figure 5:**
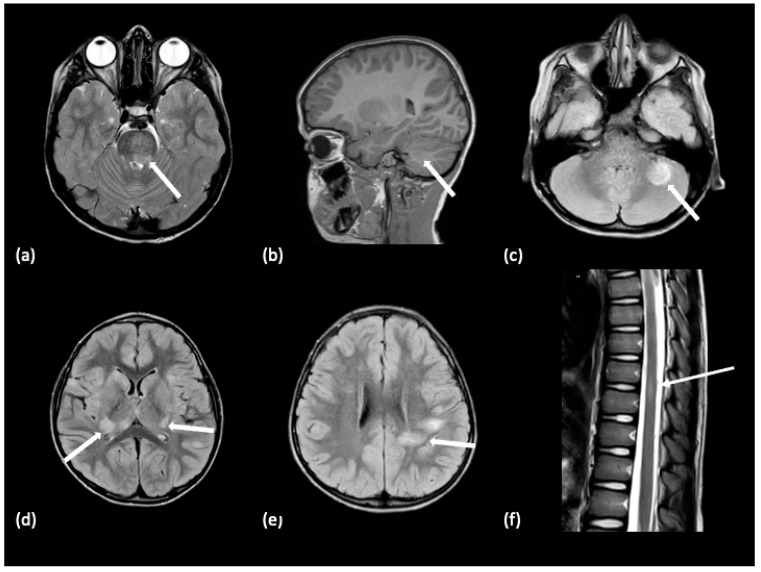
A 7-year-old male presented with an acute history of fever, ataxia, and dysarthria. Brain MRI demonstrated multiple hyperintense signals on T2/FLAIR sequences that displayed faint hypointense signals on T1-weighted sequences, without diffusion restriction, abnormal contrast agent enhancement or mass effect. Lesions are noted with white arrows. (**a**) Lesions within the pons and cerebral peduncle are hyperintense on an axial T2-weighted image. (**b**) Sagittal T1-weighted and (**c**) axial FLAIR feature a lesion in the left cerebellar hemisphere. (**d**,**e**) Axial FLAIR images demonstrate bilateral thalamic and left centrum semiovale lesions, respectively. (**f**) An additional ill-defined, T2-mildly hyperintense lesion was noted at the T9–10 of the spinal cord. This lesion was without abnormal enhancement on T1-weighted imaging. These imaging features supported the diagnosis of acute demyelinating encephalomyelitis (ADEM).

Characteristic MRI findings for ADEM include large, bilateral, and poorly demarcated hyperintense signals on T2 and FLAIR sequences in the cerebral white matter and the spinal cord [74,77,79]. Lesions are typically asymmetric, variable in size, and larger than 2 cm [76,79,88]. They have not been consistently found to display gadolinium contrast agent enhancements or exhibit a mass effect [73,74,75,79,86]. If contrast enhancement is present, most lesions are simultaneously involved [86]. ADEM frequently affects the deep gray matter (thalamus and basal ganglia) symmetrically and can have involvement in the cerebellum and brainstem in about 50% of cases [19,73,74,86]. Figure 5 illustrates typical brain lesions found in the setting of ADEM involving the pons, cerebral peduncle, cerebellum, thalamus, and centrum semiovale. Lesions in ADEM tend to be at the same temporal stage of formation, which is consistent with the typical monophasic trajectory [88]. Unlike MS, ADEM does not exhibit black hole lesions on T1 but may exhibit faint T1 hypointensity [80]. Other features that distinguish the lesions found in patients with ADEM in contrast to those of MS include the sparing of lesions in periventricular areas, the lesion borders are not well-defined, the absence of Dawson’s fingers, and the resolution of lesions in follow-up MRI [80,89,90,91,92].

### 3.3. Advanced MRI Techniques

Pediatric studies using advanced MRI techniques to evaluate lesions in patients with ADEM often compare the quantitative features with those found in patients with POMS. MRS has demonstrated decreased NAA and increased Cho, lactate, and lipid concentrations in pediatric ADEM lesions; however, these findings are non-specific and have also been associated with MS [81,93,94,95]. Ben Sira et al. elucidated metabolite differences in ADEM patients at two distinct timepoints: during the acute phase (the first 12 days) and chronic phase (after 12 days) [96]. Levels of mI, an inflammatory glial cell marker, were low in the acute phase and later increased in the chronic phase [96,97], a distinguishing feature from MS lesions, which maintain elevated mI levels irrespective of the time point [39,45,98]. Therefore, fluctuations in levels of ml can help differentiate between ADEM and initial presentations of POMS [60,96].

Additionally, DWI is used to describe the pathological evolution of pediatric ADEM, though there is conflicting evidence surrounding diffusivity patterns in ADEM. Vasogenic edema, signified by isointense or hyperintense lesions on DWI and hyperintense lesions on ADC mapping, is observed in 75% of patients [99]. In pediatric ADEM, it has been found that ADC values from DWI are initially high but decrease over time after symptom onset [99]. Together, these findings suggest that vasogenic edema may be involved in early disease pathophysiology and contribute to the overall favorable disease prognosis, as vasogenic edema is reversible [99]. A study by Chen et al. assessing a 17-year-old pediatric patient with ADEM with DTI found significantly elevated ADC and radial diffusivity (RD), along with reduced FA values in the subacute stage, with biopsy confirmation of active inflammatory demyelination [100]. RD is a diffusion metric thought to reflect changes specific to the myelin sheath. Balasubramanya et al. reported a cross-sectional study of eight patients with ADEM lesions who demonstrated low mean ADC values in the acute stage yet high ADC values in the subacute stage [81].

While MTR values did not differ in adult patients with ADEM and healthy controls, normal-appearing brain matter MTRs were significantly higher in patients with ADEM compared to MS, indicating the utility of MTI in distinguishing the two diseases [101]. Thus, MTI indicates the sparing of the pathologic process in patients with ADEM, which supports the overall favorable prognosis and recovery of lesions after the initial episode of ADEM [101].

### 3.4. Summary of Imaging in Pediatric Patients with ADEM

ADEM can occur as a monophasic, immune-mediated condition in children following a prior infection or vaccination and demonstrate large, asymmetric lesions with poorly established borders involving both white and gray matter, including the centrum semiovale, thalamus, cerebellum, and brainstem. The lesions follow the same time course in evolution, but do not typically uptake gadolinium contrast agents. Advanced imaging techniques often quantify lesion properties, support reversible pathophysiology, and discriminate from POMS.

## 4. Optic Neuritis

### 4.1. Overview

ON involves injury to the optic nerve due to inflammatory, infectious, or autoimmune causes. ON may be part of acquired demyelinating syndromes and a cluster of diseases with various clinical and radiological features, serologic and plasma biomarkers, and variable prognosis [102]. ON is rare in pediatric patients compared to adult patients yet accounts for 25% of pediatric demyelinating diseases [103], with an incidence range of 0.2 to 1.66 per 100,000 person-years in Canada and the United States, respectively [104,105,106]. ON can occur idiopathically as a clinically isolated syndrome or as a manifestation of underlying relapsing conditions, such as MS, NMOSD, and MOGAD [103,106]. The average age of onset in pediatric patients with ON is between 9 and 11 years [103,106]. ON presents differently in children compared to adults. Pediatric presentation is characterized by severe visual symptoms, including poor visual acuity, acute or subacute vision loss, double vision, and color vision deficits [103,106]. Pain with eye movement is seen in almost every adult with ON but was reported in only approximately half of pediatric patients with ON [107,108]. Children are more likely to have bilateral ON, and the rate of bilaterality is age-dependent: 72% of children under 10 years have bilateral presentation, while 70% of children over 10 years are affected in one eye [109,110]. Pediatric ON is also more likely to exhibit papillitis over retrobulbar involvement, optic disc edema or hemorrhage, and pupillary defects compared to adult ON [103,106,111,112]. Despite extreme visual disturbance at presentation, pediatric ON has a generally positive visual prognosis, with 70–85% of eyes returning to 20/20 vision [113].

Imaging features may be able to assist in predicting which patients presenting with ON may progress to have relapsing demyelinating diseases. Children with ON have a 36% risk of developing MS within two years [112]. Risk factors associated with developing MS are the presence of bilateral ON and white matter lesions outside of the optic nerve [111,112,114]. Additional features of optic neuritis may suggest NMOSD or MOGAD. Longitudinally extensive optic nerve involvement, bilateral optic nerve involvement, and chiasmal involvement seen on imaging in pediatric ON is associated with NMOSD and may suggest an increased risk of demyelinating attacks [115]. This contrasts with MOGAD-associated ON, which is classically bilateral, longitudinally extensive, associated with significant edema, more anterior predominant, and involving the optic nerve sheath [87,116,117,118,119,120].

### 4.2. Conventional Neuroimaging

**Figure 6:**
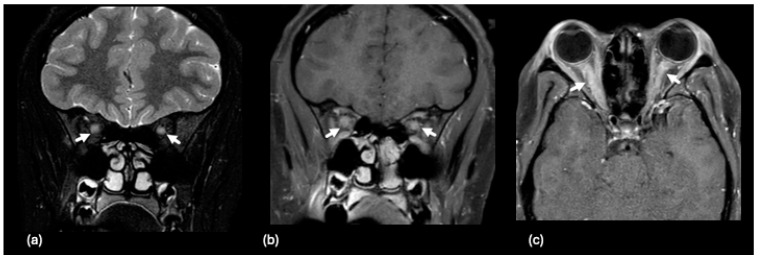
A 13-year-old female presenting with bilateral papilledema and imaging findings of optic neuritis. Coronal T2-weighted image (**a**) with fat saturation demonstrates bilateral optic nerve edema and swelling (white arrows). Coronal (**b**) and axial (**c**) fat-saturated, post-contrast T1-weighted images demonstrate abnormal enhancement (white arrows). The authors formally obtained permission to use clinically generated medical images stored within the Cincinnati Children’s Hospital Medical Center’s PACS for this figure on 6 December 2024.

The routine imaging protocol for the optic nerve employs coronal T2-weighted imaging with fat suppression and pre- and post-contrast axial and coronal T1-weighted imaging without fat suppression, as illustrated in Figure 6 [121]. Incorporating three-dimensional sequences minimizes partial volume effects, where tissue boundaries are blurred from inadequate spatial resolution, though acquisition times can be longer unless acceleration schemes in sequence software are employed to reduce acquisition times [121]. FLAIR and diffusion sequences are also helpful. In general, imaging findings indicate unilateral or bilateral enlargement, abnormal signals, and/or enhancement of the optic nerve [112,122]. The first occurrence of symptoms may be associated with a range of optic nerve swelling on imaging that may progress to optic nerve atrophy over time [122]. Similarly, during the acute presentation of ON, imaging may reveal optic nerve enhancement on post-contrast T1-weighted imaging, which is absent in the chronic phase [122]. The reported prevalence of optic nerve enhancement in pediatric patients with ON varies greatly, and in two studies, it ranged from 29 to 81% [123,124]. The wide discrepancy is likely attributed to variations in patients or differences in imaging protocols and performance quality. Pediatric patients with ON may also experience white matter lesions external to the optic nerve, a finding that has been described in 38–54% of cases [111,112].

### 4.3. Advanced MRI Techniques

Many advanced neuroimaging techniques exist for imaging the optic nerve, though they are typically described in adult populations. Future research efforts are needed to implement advanced orbital MRI into evaluations of pediatric ON. Improved visualization will provide diagnostic benefits along with therapeutic monitoring and prognostication. The fat-suppressed T1-weighted three-dimensional radial gradient-recalled echo-volumetric interpolated breath-hold examination (Radial VIBE) is often used to produce images with better quality than a typical T1, T2, or FLAIR sequence [121]. Additionally, half-Fourier acquisition single-shot turbo spin echo (HASTE) imaging can be useful for determining the diameter of the optic nerve [121]. DWI can be helpful in assessing the acuity of the attack, as most patients with acute ON exhibit DWI hyperintensity of the optic nerve, which is not as commonly found in chronic ON [122]. Additionally, ADC values are lower in patients with acute ON compared to those with chronic ON [122]. This can also be helpful in predicting outcomes, as decreased ADC values at the acute stage of attack correlate with the thinning of the retinal nerve fiber layer and ganglion cell complex and have implications for predicting optic nerve atrophy [107].

### 4.4. Summary of Imaging in Pediatric Patients with ON

ON can occur as a clinically isolated syndrome or as a manifestation of an underlying relapsing condition. Children with ON demonstrating bilateral involvement and brain white matter lesions are at greater risk of relapsing demyelinating disease. There is a need for improved MRI techniques to better characterize orbital pathology in pediatric patients, as assessing the disease stage and responses to treatment and prognostication are beneficial, especially as children may not experience pain and be unable to describe vision changes.

## 5. Neuromyelitis Optica Spectrum Disorders

### 5.1. Overview

NMOSD is a neuroinflammatory disease reported to have an incidence of 0.039 to 0.73 per 100,000 person-years for adults and 0.01 and 0.06 per 100,000 person-years for children [125]. Childhood-onset NMOSD has an average onset age of 10 years [126] and a lower female:male ratio (3:1) than adult NMOSD (9:1) [127,128,129]. Immunoglobin G autoantibodies (IgG) against the aquaporin 4 (AQP4) channel in patient serum serve as specific biomarkers for NMOSD [130,131] and is a diagnostic criterion for formal antibody-positive NMOSD diagnosis [129]. Aquaporin is a protein molecule expressed in astrocyte foot processes to regulate the passage of water across cellular membranes [130]. Until the discovery of the AQP4-IgG, NMOSD was not considered its own disease entity but a subtype of MS. Patients with clinical criteria meeting NMOSD diagnosis but with seronegative AQP4 findings are designated antibody-negative NMOSD [129]. Chitnis et al. found that approximately 65% of pediatric NMOSD patients tested positive for the AQP4 antibody [126], while prior studies reported a wider range of 17–80% [113]. NMOSD is characterized by attacks of ON and transverse myelitis, owing to various visual, motor, and sensory symptoms [132,133,134,135]. ON is more often seen in children’s initial presentation, though transverse myelitis frequently occurs in relapses [135,136,137]. Other common symptoms in children include severe and intractable nausea, hiccups, and vomiting due to the involvement of the area postrema [135,138]. Pediatric patients with NMOSD also experience encephalopathy at higher rates than their adult counterparts [135]. Acute brainstem syndrome, causing cranial nerve dysfunction, occurs in 40% of pediatric NMOSD patients [127]. Patients with NMOSD may also present with seizures, headaches, and ataxia [132,133,135,139]. Early diagnosis is crucial for NMOSD, as delays in treatment can have severe consequences and result in disability [132,140].

### 5.2. Conventional Neuroimaging

**Figure 7:**
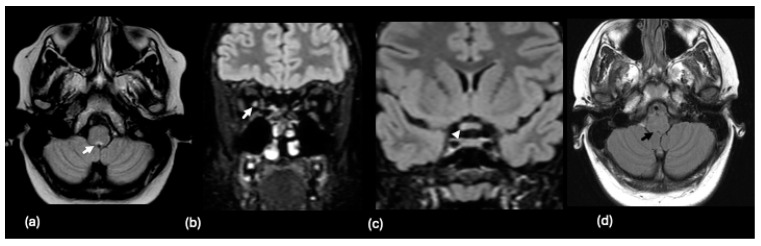
A 10-year-old male presenting with left lower limb weakness, pain, and hyperreflexia. (**a**) Axial FLAIR image demonstrates a non-enhancing, small, focal hyperintense signal abnormality along the posterior inferior medulla (white arrow). On coronal FLAIR (**b**,**c**), there is asymmetric edema in the right optic nerve (white arrow) and subtle swelling and signal abnormality of the right optic chiasm (white arrowhead). (**d**) 2-year-follow-up MR shows resolution of medullary signal abnormality (black arrow) on axial FLAIR.

Brain lesions are present in approximately 30–40% of pediatric patients with NMOSD [141,142,143]. The location of lesions is similar in both pediatric and adult populations with NMOSD, but disease involvement in the brain is more common in pediatric patients [132]. Lesions often appear in areas where there is high AQP4 expression, specifically near the third and fourth ventricles in the diencephalon, area postrema, and brainstem, as illustrated in Figure 7 [132]. Many studies have found brain lesions in the corpus callosum, subcortical white matter, and periventricular white matter, as well as in the thalamus and hypothalamus [132,144,145]. Like adults with NMOSD, about 30% of pediatric patients with NMOSD brain lesions exhibit a distinctive cloud-like pattern of gadolinium contrast agent enhancement [132,141,144]. A linear, pencil-thin enhancement of the ependymal surface of the lateral ventricles has also been described as characteristic of NMOSD [141,146,147]. Corpus callosum lesions in NMOSD are large and irregularly shaped, following the ependymal line [147]. This contrasts with the smaller, ovoid lesions (Dawson’s fingers) typically seen in MS [147]. Patients with NMOSD are also less likely to develop silent lesions, a common finding in MS [147]. Large or tumefactive confluent lesions in the white matter (>3 cm) without a mass effect are more common in pediatric than adult patients with NMOSD [113,147,148].

**Figure 8:**
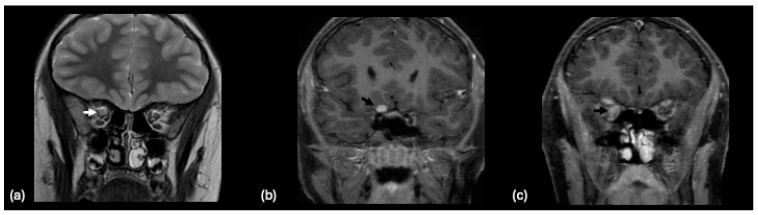

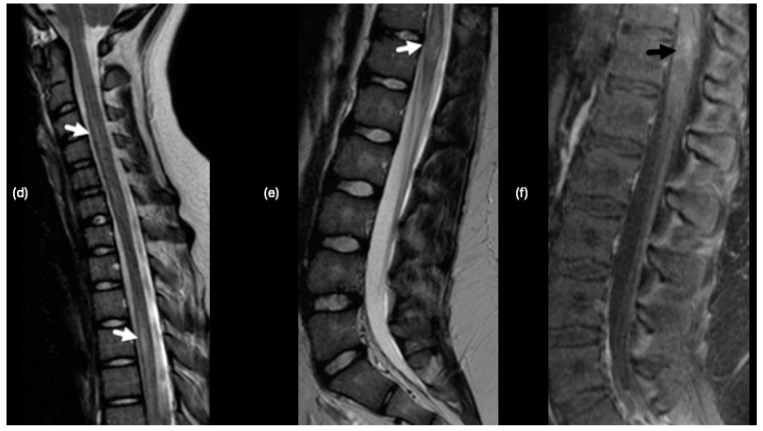
A 10-year-old female presenting with right monocular vision loss. (**a**) Coronal T2 orbital image demonstrates abnormal edema and swelling of the intraorbital right optic nerve (white arrow), as well as abnormal enhancement (black arrows) of the (**b**) prechiasmatic (**b**,**c**) intraorbital right optic nerve. Six weeks later, the patient presents with new voiding and extremity weakness. On (**d**,**e**) sagittal T2-weighted images, there are ill-defined cord signal abnormalities (white arrows) with associated subtle enhancement (black arrow) of the distal cord on (**f**) sagittal T1-weighted post contrast fat saturated spine MR image.

The classic presentation of optic nerve involvement associated with NMOSD on MRI is bilateral inflammation of the posterior optic nerves (optic chiasm and optic tracts) [132,133,144,149], as illustrated in Figure 8a–c. A special imaging feature specific to the pediatric presentation of NMOSD is infraorbital fat gadolinium contrast agent enhancement for patients with ON [132,150].

In spinal cord imaging, longitudinally extensive transverse myelitis (LETM), appearing on T2 images as central hyperintensity over three or more vertebral segments, usually indicates NMOSD in adults. However, in children, LETM is a less-specific indicator for NMOSD, as ON tends to occur more often prior to the age of 30 [132,136,151,152]. An example of abnormal spinal imaging in a child with NMOSD is illustrated in Figure 8d–f.

### 5.3. Advanced MRI Techniques

Because NMOSD primarily affects adults, with only 3–5% of patients with NMOSD being children [132,135], the data on advanced neuroimaging techniques in pediatric cases are sparse. A review by Kremer et al. describes advanced neuroimaging in adult patients with NMOSD [153]. There is not sufficient evidence that MRS is sensitive to NMOSD, as NAA, creatine, and choline levels are reported as within normal levels. DTI for adults with NMOSD shows that non-lesional damage more often occurs in the connecting tracts around lesions [154,155]. Spinal lesions in NMOSD showed higher RD than patients with MS, reflecting more severe tissue damage [155], which correlates with the poor relapse recovery noted in these patients [155]. Other studies have indicated extensive white matter damage through DTI, noting significant FA decreases in the pyramidal tract, optic radiation, and corpus callosum in patients with NMOSD [156]. Abnormalities in the NAGM, especially the thalamus and putamen, with an increased average FA compared to age- and sex-matched healthy adults, have also been revealed through DTI [157]. Increased ADC in NMOSD lesions reflects vasogenic edema during acute inflammation [147]. In the NAGM of patients with NMOSD, MTI revealed tissue damage upon finding lower MTR histogram metrics and increased MD on DTI; however, the MTR values in NAWM were within normal ranges [158].

### 5.4. Summary of Imaging in Pediatric Patients with NMOSD

NMOSD is a rare autoimmune inflammatory demyelinating disease in children. Pediatric patients present with more brain involvement than adults. Given the distinct antibodies (AQP4-IgG) of NMOSD, lesions observed on conventional MRI tend to appear in brain regions with AQP expression. Future investigations with advanced MRI techniques, especially diffusion imaging, in pediatric patients with NMOSD could offer more information about the extent of disease involvement, and guide management in efforts to reduce inflammation and demyelination, given the severe consequences if not treated early with immunotherapy.

## 6. Myelin Oligodendrocyte Glycoprotein Antibody Disease

### 6.1. Overview

MOG antibodies have long been recognized in patients with demyelinating syndromes; however, MOGAD has recently been recognized as a distinct disease entity with a unique pathology and prognosis [87,159]. MOGAD is rare, with a higher incidence in children (3.1 per million) than adults (1.3 per million) [117,160]. MOGAD can occur on its own, or MOG antibodies can be detected in other demyelinating syndromes, such as NMOSD, but are extremely rare in MS [87]. MOG is a CNS-specific protein and is expressed on oligodendrocyte surface membranes and on the outer layer of the myelin sheath, rendering it an ideal target for immunogenic antibodies in demyelinating processes [116]. Patients diagnosed with MOGAD are seropositive for an antibody against MOG and exhibit a variety of symptoms similar to other neuroinflammatory and demyelinating diseases [116,117,119,161]. Pediatric MOGAD manifests with both monophasic or relapsing courses, typically presenting with ADEM (46%), ON (30%), transverse myelitis (11%), or an NMOSD-like phenotype with ON and transverse myelitis [117]. Brain involvement, as in ADEM, is seen more frequently in young children, whereas ON and spinal involvement is seen more in older children and adult patients [159]. Additionally, cerebral cortical encephalitis, brainstem, and cerebellar involvement, leading to fever, headache, nausea, vomiting, seizures, visual impairments, motor deficits, and cognitive impairment, are also described in the literature [116,117,161].

### 6.2. Conventional Neuroimaging

**Figure 9:**
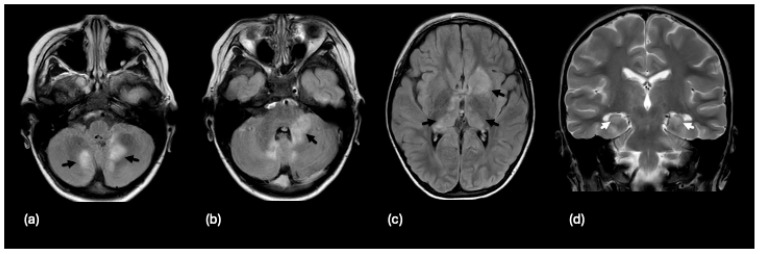
A 4-year-old male with recent viral encephalitis presenting with new gait ataxia, finger tingling, and headache and positive MOG antibody. Axial FLAIR images demonstrate multifocal, non-enhancing lesions (black arrows) in the (**a**) cerebellum, (**b**) left brachium pontis, and (**c**) deep gray matter structures, including the left caudate, anterior left putamen and globus pallidus, and the right greater than left thalami. Additional bilateral hippocampal swelling and signal abnormalities (white arrows) are shown on (**d**) coronal T2-weighted images.

Neuroimaging findings in pediatric patients with MOGAD are heterogeneous throughout the brain and depend on the clinical manifestation. White matter lesions in children with MOGAD often follow a large and confluent pattern, “leukodystrophy-like” [118,162]. More specifically, the literature has reported T2 hyperintense lesions in the corpus callosum, orbital frontal gyrus, thalamus, basal ganglia, cerebellar peduncles, and brainstem adjacent to the fourth ventricle [117,118,119,120]. An example is illustrated in Figure 9.

In MOGAD-associated ADEM, imaging may reveal large, poorly demarcated T2 hyperintense lesions in the bilateral supratentorial and subcortical and deep white matter as well as deep gray matter [118,120]. In MOGAD-associated NMOSD, periventricular lesions are more typically found along the third ventricle as well as in the periaqueductal grey matter and dorsal brainstem. Confluent white matter lesions in the juxtacortical white matter and cortical and deep grey matter lesions are found in patients with MOGAD-associated autoimmune encephalitis [118].

Optic nerve swelling is observed but appears to be poorly demarcated. MOGAD typically involves the anterior segment of the optic nerve and spares the optic chiasm, unlike NMOSD, which often involves the chiasm and posterior segment [117,118,119,120]. In the spinal cord, common imaging findings include LETM spanning across three or more vertebral segments, frequently in the cervical and thoracic spine [117,118,119,120].

While imaging features of MOGAD seem non-specific, there are a few distinguishing factors on MRI. Curvilinear corpus callosal lesions and poorly demarcated lesions are seen more frequently in young children with MOGAD compared to other demyelinating diseases [118]. Patients with MS can have low-titer MOG antibodies, which can make it difficult to differentiate between these two entities, but the presence of Dawson’s Fingers, inferior temporal lobe lesions, lesions within subcortical U fibers, ovoid lesions perpendicular to the lateral ventricle, and short segment spinal cord lesions are often indicative of MS rather than MOGAD [117,118,120].

### 6.3. Advanced MRI Techniques

As for more advanced MRI techniques, DTI and quantitative susceptibility mapping have been described in the literature; however, this is mostly for adult MOGAD patients. Automated fiber quantification (AFQ) allows for a more detailed analysis and localization of abnormalities in white matter tracts using DTI metrics [163]. AFQ technology in pediatric MOGAD patients has shown widespread FA reductions and RD elevation in white matter tracts, indicating disruption of white matter microstructure and myelin sheath integrity, respectively [163]. Potential MOGAD biomarkers significantly associated with an expanded disability status scale include the FA of the left cingulum cingulate and the RD of the right inferior frontal-occipital fasciculus [163]. In a study by Song et. al. DWI was performed on pediatric patients with MOGAD, but none presented with diffusion restriction [161]. ^31^P-Phosphorus MRS detects adenosine triphosphate (ATP), phosphocreatine, and inorganic phosphate levels and reflects energy metabolism in the brain. Specifically, β-ATP peaks correspond to intracellular free magnesium (Mg^2+^) levels [164]. Pediatric patients with MOGAD exhibit β-ATP peak splitting, resulting in two distinct peaks. MOGAD patients demonstrate low intracellular Mg^2+^ levels in white matter areas and more acidic pH in the brain [164]. Mg^2+^ plays a critical role in ATP metabolism, and these observations suggest that oligodendrocytes in the white matter of patients with MOGAD are susceptible to Mg^2+^ deficiency [164].

### 6.4. Summary of Imaging in Pediatric Patients with MOGAD

The pediatric presentation of MOGAD is more common, and the imaging is more heterogeneous, as patients with other demyelinating diseases are also MOG-IgG positive. Analogous to ON and NMOSD, the lack of advanced imaging investigations for pediatric populations with MOGAD needs to be addressed in the future with multisite longitudinal studies to overcome limitations of small numbers of patients at a single institution and co-occurrence with other demyelinating diseases. Diffusion imaging sequences are routinely implemented in clinical settings and would be the most amenable for quantitative analyses in future therapeutic trials.

## 7. Autoimmune Encephalitis

### 7.1. Overview

Autoimmune encephalitis is another antibody-mediated neuroinflammatory condition with an estimated incidence of 1.54 to 2.2 per million children per year based on two studies [165,166]. Children with autoimmune encephalitis are previously healthy, with prodromal symptoms including a fever and headache that precedes a rapid onset of symptoms, such as seizures, movement disorders such as ataxia, dystonia, chorea, myoclonus, or tremors, disturbed sleep, agitation, altered levels of consciousness with some degree of cognitive impairment, and confusion [167]. Children experience neurological manifestations (seizure or movement disorders) more often than the psychiatric (anxiety or paranoia) and autonomic symptoms seen in adolescents and adults [167,168]. As opposed to a condition like MOGAD that involves a single type of antibody, autoimmune encephalitis can involve many different antibodies, resulting in different subtypes of autoimmune encephalitis. The most common type is anti-N-Methyl-D-Aspartic receptor (NMDAR) encephalitis, which is the focus of case studies [169,170]. NMDAR encephalitis is more common in females in patients older than 12 years [167,169]. In some patients with NMDAR encephalitis, immunogenic antibodies are triggered by prior herpes simplex viral encephalitis or an ovarian teratoma [165]. However, children are much less likely to have an associated teratoma compared to adults [167,168]. Pediatric patients have a good prognosis but slow recovery, with 85% following a monophasic course [171].

### 7.2. Conventional Neuroimaging

**Figure 10:**
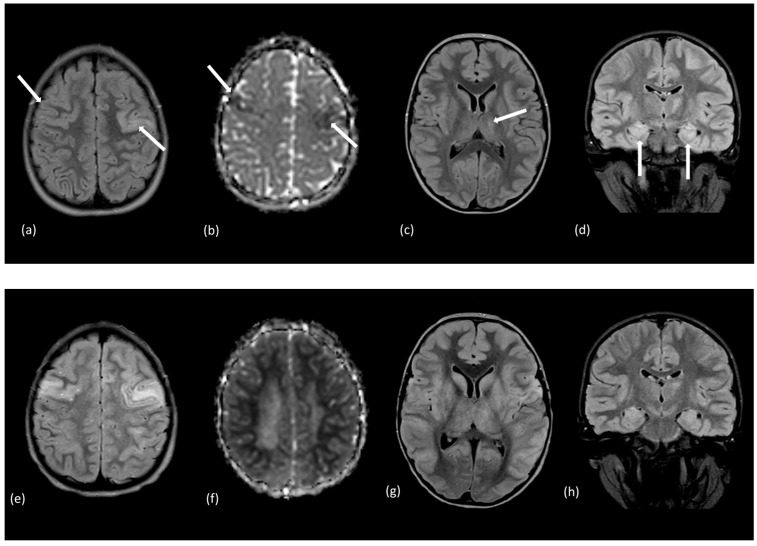

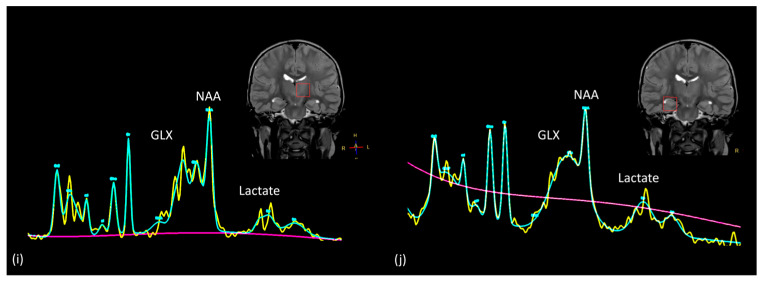
A 6-year-old male presented with new onset seizures, facial droop, and a family history of transient ischemic attacks. The initial MRI with (**a**) axial FLAIR image and (**b**) diffusion ADC map demonstrated multifocal areas of abnormal signal bilaterally (white arrows) involving the superior frontal gyri. Axial FLAIR imaging also revealed (**c**) a left thalamic lesion, and (**d**) hippocampal formations showing increased signal intensity and enlargement without diffusion restriction. Select FLAIR images from a follow-up study performed a week later showed progression throughout (**e**) frontal cortices, (**g**) thalamus and insular cortices, and (**h**) hippocampi. The (**f**) ADC map demonstrated restriction throughout subcortical white matter. Additional lesions emerged in the parietal lobes, pons, cerebellar white matter, thalami, and basal ganglia. MRS (**i**,**j**) with voxel placement illustrated on coronal T2 images featured elevated signal for lactate (1.3 ppm) and glutamate/glutamine (GLX, 2.1–2.5 ppm) accompanied by reduced N-acetyl aspartate (NAA) (2.0 ppm) levels for the left thalamic and right hippocampal regions sampled. These features supported the diagnosis of autoimmune encephalitis.

Demyelination is not a common feature of autoimmune encephalitis and only occurs in 8.7% of pediatric patients with NMDAR encephalitis [172]. Atypical MRI findings on T2-weighted and FLAIR sequences are found in less than half (31–40%) of pediatric patients with autoimmune encephalitis [168,171,173,174,175,176]. NMDAR encephalitis imaging findings are diverse and manifest in all lobes, especially frontal, temporal, and parietal, and include cortical, subcortical, basal ganglia, and infratentorial T2 hyperintense lesions [167,171,175]. T2 hyperintense frontal and occipital lesions are associated with poor outcomes and residual neuropsychological dysfunction [175]. Meningeal enhancement may be seen in contrast-enhanced MRI [175]. Imaging may evolve over time, and some children who have normal initial MRI show brain atrophy, with ventricular enlargement at the follow-up examination [176]. Other imaging features that may suggest specific antibodies associated with autoimmune encephalitis include increased T2/FLAIR signals in the mesial temporal lobe, which is observed in rarer encephalitides with leucine-rich glioma-inactivated 1 and contactin-associated protein-like 2 antibodies [171]. Recognition of imaging findings may guide medical management in supporting the initiation of immunotherapy and minimizing procedures and treatments that have low benefits [177]. Figure 10a–h depicts the imaging of a pediatric patient with autoimmune encephalitis who was found to demonstrate hyperintense signals in the thalamus, hippocampi, and frontal gyri. Figure 10i,j demonstrates MRS in a patient with autoimmune encephalitis, with an increase in the lactate and glutamate/glutamine levels and a decrease in the NAA levels.

### 7.3. Advanced MRI Techniques

Given the different antibodies, heterogeneous findings have been reported in the literature, with advanced MRI techniques from a few dedicated pediatric investigations. DWI appearances may vary in patients with autoimmune encephalitis. Armangue et al. detailed a pediatric patient with herpes simplex encephalitis followed by NMDAR encephalitis, in which the initial DWI displayed increased signal and restricted diffusion in the opercular regions that progressed to encephalomalacia one month later [169]. This finding does not seem to be universal, as the type of autoimmune encephalitis often affects the presence or absence of diffusion restriction. Diffusion restriction may occur in response to swelling, seizure activity, inflammatory infiltration, gliosis, and neuronal loss [178]. Another study by Kotsenas et al., investigating MRI characteristics in patients with autoimmune voltage-gated potassium channel complex encephalitis with seizures, found restricted diffusion in mesial temporal lobe structures such as the hippocampi [177]. A study by Lascano et al. reported the absence of diffusion restriction in GABA_A_R autoimmune encephalitis [174]. Finke et al. used DTI to analyze white matter in patients with NMDAR encephalitis and found an overall reduction in FA, an increase in MD, and RD localized to the cingulum [179].

Cai et al. conducted a resting state fMRI study evaluating adult and pediatric patients with NMDAR encephalitis [180] and found a reduced amplitude of low-frequency fluctuation values bilaterally in the cerebellum and posterior cingulate gyrus as well as the left precuneus. Additionally, it was shown that the lingual gyrus, posterior cingulate gyrus, fusiform gyrus, calcarine, cuneus, and posterior central gyrus displayed increased FC [180]. These findings may contribute to cognitive and emotional deficits observed in patients following recovery from NMDAR encephalitis.

### 7.4. Summary of Imaging in Pediatric Patients with Autoimmune Encephalitis

Children with autoimmune encephalitis are previously healthy, with prodromal symptoms, including fevers and headaches that precede a rapid onset of neurological symptoms. Conventional imaging may be absent specific findings for most pediatric patients evaluated for autoimmune encephalitis. However, some pediatric patients with initially unremarkable imaging may go on to develop volume loss that is observed in follow-up imaging examinations. The inability of conventional MRI to display any features in these patients speaks to the critical need for advanced imaging techniques to reveal occult pathological processes. The findings of diffusion restriction are non-specific as they represent multiple processes, but this technique does hold the potential for increasing the sensitivity of the detection of regional involvement from autoimmune encephalitis with MRI. For those with imaging findings, increased signal intensity on T2-weighted and FLAIR imaging throughout the brain reflect the inflammatory processes that occur and are associated with the immune response.

## 8. Febrile-Infection-Related Epilepsy Syndrome

### 8.1. Overview

FIRES is perhaps the most elusive and terribly fatal of the neurologic conditions detailed in this review. This syndrome occurs mostly in children and is rare, affecting approximately 1 in 1,000,000 children [181]. Unfortunately, there is not a clear etiology to FIRES; it is unknown if the etiology is infectious, autoimmune, or a combination of the two, and biomarkers are largely non-specific [181,182,183]. However, prior febrile infection is a diagnostic requirement for FIRES and may explain its origin to some degree [181,182,183]. Often, children are infected with an upper respiratory infection or gastroenteritis before initial manifestations of FIRES [181,182,183]. There are two recognized phases: acute and chronic. The acute phase is often characterized by fever, recurrent seizures, vomiting, liver dysfunction, arrhythmia, headaches, drowsiness, confusion, and skin rash [181,182,183]. In the chronic phase, seizures evolve into status epilepticus, causing neurological, neuropsychological, and cognitive impairment [181,182,183,184,185].

### 8.2. Conventional Neuroimaging

**Figure 11:**
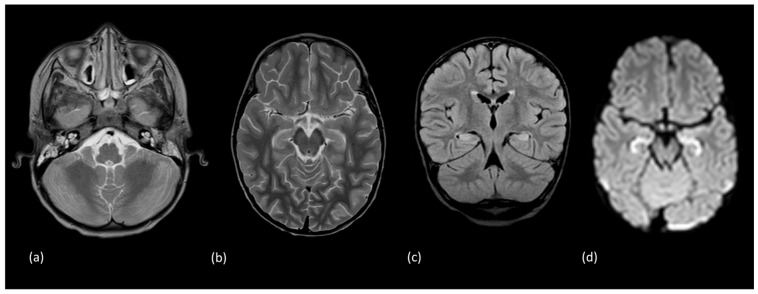

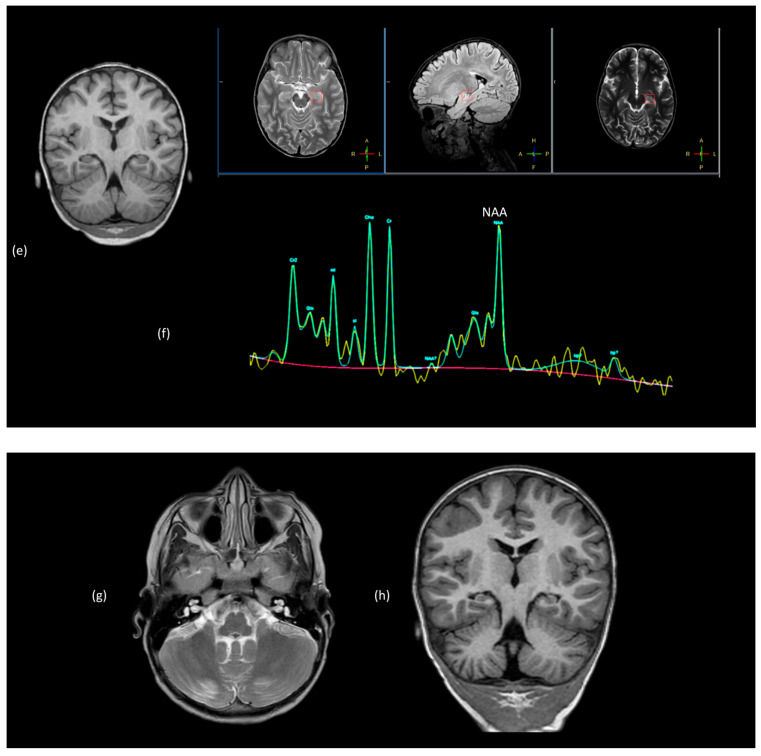
A 3-year-old male presented with acute onset seizure. Initial MRI demonstrated increased signal intensity and diffusion restriction involving both hippocampal formations with subtle signal abnormality involving the adjacent amygdala. (**a**) Axial T2-weighted imaging at the level of the cerebellum. (**b**) Axial T2-weighted, (**c**) coronal FLAIR, and (**d**) axial diffusion-weighted imaging reveal hyperintense signal within the hippocampus. (**e**) Initial coronal T1-weighted imaging. (**f**) Short echo magnetic resonance spectroscopy of the left hippocampus demonstrated reduced N-acetyl aspartate (resonance located at 2.0 ppm). In a follow-up study performed 6 months later demonstrated resolution of the noted hippocampal signal findings. However, asymmetric volume loss of the cerebellum with prominent cerebellar sulci ((**g**) axial T2-weighted) and right hippocampal formation ((**h**) coronal T1-weighted) imaging. These features were consistent with a diagnosis of febrile-infection-related epilepsy syndrome (FIRES).

This syndrome exhibits differing neuroimaging findings associated with the phase of disease. In the acute phase, over half to nearly two-thirds presented with an unremarkable MRI [186,187,188]. For those with findings, hyperintense lesions in the temporal lobe, hippocampi, and insular cortex are more commonly observed [187,188]. Leptomeningeal enhancement may be found as well [186,188]. Acute presentation with hippocampal hyperintensity is illustrated in Figure 11.

The chronic phase of FIRES is largely characterized by atrophy, which is likely associated with the neurocognitive decline observed in the late phase. More specifically, studies have reported ventriculomegaly, mesial temporal lobe, and cerebellar atrophy [187,188]. Other distinguishing neuroimaging features of FIRES include deterioration and subsequent sclerosis of the hippocampus (typically within 15 days of seizure onset), followed by widespread brain atrophy (typically within a month after onset) [187]. A study by Lee et al. also suggests that more extensive focal lesions with increased T2 and FLAIR signals within the periventricular white matter during the acute phase may predict an overall worse clinical outcome [183].

### 8.3. Summary of Imaging in Pediatric Patients with FIRES

The etiology of FIRES is not currently defined; however, prior febrile infections remain a requirement for making a diagnosis. Acute-phase conventional imaging may not demonstrate any abnormalities. However, for pediatric patients with findings, lesions with hyperintense signals are observed in the hippocampi and insular cortex. In the chronic phase, like autoimmune encephalitis, undetected processes are occurring that result in volume loss in the brain, which is an urgent unmet need for research investigation to develop methods to uncover the etiology and map the extent of involvement within the brain using imaging techniques.

## 9. Conclusions

This review provided a broad overview of demyelinating and neuroinflammatory diseases that present in children. Currently, the most sensitive neuroimaging tools to help clinicians in diagnosing and characterizing these diseases in the brain and spine are conventional magnetic resonance imaging sequences that include multiplanar, standard T1-weighted (with and without contrast), T2-weighted, and FLAIR sequences. A comparative table summarizing conventional imaging features for these diseases is presented in Table 1. Patterns have emerged that can assist in narrowing the diagnostic differential. Advanced MRI-based techniques for POMS and other neuroinflammatory diseases may be helpful in differentiating these disorders and serve as biomarkers for disease progression. However, the widespread implementation of these techniques has been limited due to several factors, including the rarity of patients with these diseases, restrictions imposed on the length of imaging studies due to the acute morbidity of this patient population, and a lack of widespread availability of these techniques within the clinical research setting and accompanying expertise in acquisition and interpretation. As many of these advanced MRI techniques become standardized across scanner vendors and institutions, it will be imperative for clinicians, neuroradiologists, and imaging researchers to implement multisite investigations to identify imaging biomarkers in patients with demyelinating diseases and other neuroinflammatory conditions with the goal of obtaining objective biomarkers for therapeutic trials.

## Figures and Tables

**Table 1:** Comparative summary of Conventional MRI features of pediatric-onset multiple sclerosis and pediatric neuroinflammatory diseases.

Disease	Presentation	Locations of Involvement	Enhancement	Comments
Pediatric-onset Multiple Sclerosis	Two distinct events; Relapsing	Periventricular lesions in deep white matter, Juxtacortical (subcortical white matter) lesions, infratentorial lesions that are focal, ovoid-shaped lesions. Spinal cord lesions spanning less than 3 vertebral segments.	Active lesions show enhancement	Small, ovoid lesions perpendicular to long axis of corpus callosum (Dawson’s fingers); Lesions in subcortical U-fibers; Silent lesions; Black holes (T1 hypointense lesions); Variable age of lesions
ADEM	Typically, Monophasic following infection or vaccination	Centrum semiovale, thalamus, basal ganglia, cerebellum, brainstem; bilateral involvement; spinal cord involvement	Inconsistent lesion enhancement: if present, most lesions show enhancement	Asymmetric white matter, but symmetric deep gray matter lesions with borders not well-defined; variable sizes, larger than 2 cm; Similar age of lesions
ON	Acute Phase	Enlargement, swelling of the optic nerve; >70% bilateral involvement in children < 10 years	Optic nerve enhancement	May also be featured in other diseases
	Chronic phase	Atrophy of the optic nerve	Absent optic nerve enhancement	
NMOSD	At presentation: >60% unremarkable	N/A	N/A	N/A
	At presentation: <40% with findings	Corpus callosum, subcortical white matter, periventricular white matter, area postrema, brainstem, near the 3rd and 4th ventricles, thalamus and hypothalamus; Spinal cord transverse myelitis	Cloud-like pattern; Linear, pencil-thin of the ependymal surface of lateral ventricles	Corpus callosum lesions-large, irregularly shaped; Tumefactive, confluent lesions->3 cm
NMOSD with ON	At presentation: <40% with findings	Bilateral, longitudinally extensive posterior optic nerves (optic chiasm, optic tracts); spinal cord—central over three or more vertebral segments	Infraorbital fat enhancement	Initial presentation with ON; relapses with transverse myelitis
MOGAD	MOGAD	Corpus callosum, orbital frontal gyrus, thalamus, basal ganglia, cerebellar peduncles, brainstem; Spinal cord -longitudinally extensive transverse myelitis	Less common; 25% spinal	Large, confluent, leukodystrophy-like lesions; curvilinear corpus callosal lesions, lesions with borders not well-defined in young children
	MOGAD-associated ADEM	Bilateral supratentorial, subcortical white matter, deep white matter, deep gray matter (thalamus)		
	MOGAD-associated NMOSD	Periventricular lesions, periaqueductal grey matter, dorsal brainstem		
	MOGAD-associated ON	Swelling of the anterior optic nerve, optic nerve sheath; bilateral involvement		
	MOGAD-associated AE	Confluent subcortical white matter, cortical and deep grey matter lesions		
AE	At presentation: >60% unremarkable	N/A	N/A	Depends on Antibody
	At presentation: <40% with findings	NMDAR: all lobes, cortical, subcortical, basal ganglia, infratentorial	Meningeal	
FIRES	Acute phase: 2/3 unremarkable	N/A	N/A	
	Acute phase: 1/3 with findings	temporal lobe, hippocampi, insular cortex: increased T2 and FLAIR signal	Leptomeningeal	
	Chronic phase	ventriculomegaly, mesial temporal lobe, cerebellum: atrophy		

## Data Availability

Not applicable.
